# Sub-optimal gain in vision in retinal vein occlusion due to under-treatment in the real world: results from an open-label prospective study of Intravitreal Ranibizumab

**DOI:** 10.1186/s12886-020-01757-7

**Published:** 2021-01-12

**Authors:** Raja Narayanan, Aditya Kelkar, Zahir Abbas, Neha Goel, Manoj Soman, Naveen Naik, Aditya Sudhalkar, Jaydeep Walinjkar, Utkarsh Shah, Nitin Maksane

**Affiliations:** 1 L V Prasad Eye Institute, Hyderabad Eye Research Foundation, Hyderabad, Telangana India; 2 National Institute of Ophthalmology, Pune, Maharashtra India; 3 Apollo Gleneagles Heart Centre, Kolkata, West Bengal India; 4 ICARE Research Center, Noida, Uttar Pradesh India; 5 Chaithanya Eye Hospital, Trivendrum, Kerala India; 6 Narayana Nethralaya, Bengaluru, Karnataka India; 7 Iladevi Cataract & IOL Research Centre, Ahmedabad, Gujarat India; 8 Sankara Nethralaya, Chennai, Tamilnadu India; 9 Novartis Healthcare Private Limited, Mumbai, Maharashtra India

**Keywords:** Retinal vein occlusion, Ranibizumab, Macular edema, Open label prospective study, Visual acuity

## Abstract

**Background:**

Macular edema secondary to retinal vein occlusion (RVO) is an important cause of loss of vision. Intravitreal injections (IVI) of anti-vascular endothelial growth factor (VEGF) are the standard of care in this disease, as shown in numerous randomized controlled trials. The purpose of this study was to study the efficacy and safety of ranibizumab, an anti-VEGF agent, in the real-world setting.

**Methods:**

This was 48 weeks, open-label, prospective, multicentre, observational study. Patients diagnosed with ME secondary to RVO were treated with IVI of Ranibizumab 0.5 mg in real-world conditions. Efficacy was measured by improvement seen in best-corrected visual acuity (BCVA) in terms of Early Treatment of Diabetic Retinopathy Study (ETDRS) Letter Scores and change in central retinal thickness (CRT) measured by optical coherence tomography.

**Results:**

One hundred eyes of 100 patients (79 with branch retinal vein occlusion and 21 with central retinal vein occlusion) were recruited in the study. The mean (standard deviation, SD) BCVA was 52.8 (21.99) letters at baseline and 62.3 (24.40) letters at week 48. From baseline, there was a significant improvement in BCVA by 7.7 letters (*p* = 0.001) at 48 weeks. The mean (SD) of CRT was 479.9 (216.25) μm at baseline and it decreased significantly to 284.9 (171.35) μm at week 48 (*p* < 0.001). During the study period, the average number of intravitreal injections was 3.5 per patient. There was no report of endophthalmitis in any eye.

**Conclusions:**

Ranibizumab is well tolerated and effective in treating macular edema secondary to RVO in real-world clinical settings. However, there is under-treatment compared to controlled clinical trials, and the gain in vision is sub-optimal with under-treatment.

**Trial registration:**

Clinical Trials Registry - India: CTRI/2015/07/005985.

## Background

Retinal vein occlusion (RVO) is the second most common retinal vascular disease which can lead to loss of vision [1]. It is commonly of 2 types: central retinal vein occlusion (CRVO) and branch retinal vein occlusion (BRVO) [2]. Systemic comorbidities play a vital role in etiopathogenesis of RVO [1, 3]. Excessive angiogenic growth factors such as vascular endothelial cell growth factor (VEGF) caused by hypoxia secondary to RVO, leads to vascular leakage and macular edema (ME). Loss of vision is attributed to the development of ME, which occurs due to high vascular permeability caused by breakdown of blood-retina barrier [1, 4].

In a population-based study in 4711 subjects in central India, RVO was detected in 0.8% of the population. BRVO was found to be approximately seven times more common than CRVO [5].

Ranibizumab is approved by the United States Food and Drug Administration (USFDA) for the treatment of ME secondary to RVO [6]. The Drug Controller General of India (DCGI) has also approved ranibizumab for treatment of ME due to RVO.

Large randomized controlled clinical trials have substantiated the safety and efficacy of anti-VEGF agents, including ranibizumab, in treating ME secondary to RVO [7, 8]. Ranibizumab for treatment of ME following BRVO showed rapid and sustained visual improvement in patients who received monthly intravitreal injections (IVI) of 0.5 mg ranibizumab (Lucentis®, Genentech, South San Francisco, CA) [7]. At the 6-month primary end point, the mean gain in best-corrected visual acuity (BCVA) was + 18.3 letters in the 0.5 mg ranibizumab cohort compared to + 7.3 letters in the sham/laser cohort [7]. In CRUISE study, at month 12, the mean gain in BCVA was + 13.9 letters in 0.5 mg ranibizumab cohort compared to + 7.3 letters in the sham/0.5 mg cohort [8].

The efficacy and safety profile of ranibizumab demonstrated by the randomized clinical trials (RCTs) are seldom reflected in real world practice. The reasons for this variation include high internal validity, but poor external validity of RCTs, cost considerations, physician expertise, and patient factors. Compared to RCTs, real world studies include large and diverse group of patients who represent the population to which the drug is prescribed. The safety and efficacy data from such studies are more valuable for clinical practice and inform us about the gaps in outcomes.

This open label study was conducted to evaluate the effectiveness and safety of repeated IVI of ranibizumab in patients with visual impairment due to ME secondary to RVO. The findings from this study provide the real-world determinants of clinical response to ranibizumab in the Indian population.

## Methods

This was a single arm, prospective, open-label study conducted over a period of 48 weeks at 10 centers in India. Intravitreal injection of 0.5 mg ranibizumab was administered to patients with ME secondary to RVO.

The study was conducted in accordance with the Declaration of Helsinki. At each contributing study site, the conduct of the study was approved by the independent ethics committee or institutional review board, and patients provided written informed consent before participating in the study.

Patients, who were 18 years or older, were enrolled after the treating ophthalmologist made the decision of injecting ranibizumab for ME secondary to RVO. Those who had previously received either macular laser treatment or anti-VEGF therapy for RVO were also included in the study. If both the eyes were affected, only one eye was selected for the study as per the discretion of the site investigator.

Key eligibility criteria included: (1) center-involving macular edema due to BRVO and CRVO; (2) minimum central retinal thickness (CRT) of 250 μm in the central subfield on spectral domain optical coherence tomography (SD-OCT); (3) patient age of 18 years or more; (4) BCVA of perception of light to 6/9 (20/30) in the study eye.

### Exclusion criteria

Key exclusion criteria included: (1) previous anti-VEGF injection in the study eye in the last 1 month; (2) any additional intraocular disease or inflammation affecting the visual acuity (3) previous sector laser photocoagulation in the study eye; (4) any intraocular surgery in the last 1 month; (5) uncontrolled glaucoma.

### Visit schedule

A total of 9 visits were scheduled in this study. The baseline visit was followed by 6 consecutive visits, 4 weeks apart, (weeks 4, 8, 12, 16, 20 and 24). The last two visits were 12 weeks apart (week 36 and week 48).

### Baseline evaluation

Baseline ocular examinations included measurement of BCVA, slit lamp evaluation, applanation tonometry, biomicroscopy, and indirect ophthalmoscopy. All patients underwent fundus photography (Zeiss Visupac® FF4 and FF450-plus, Carl Zeiss, Dublin, CA), OCT (Carl Zeiss Meditec, Cirrus HD-OCT) and fluorescein angiography (Zeiss Visupac® FF4 and FF450-plus).

### Administration schedule of Ranibizumab

Intravitreal injection of 0.5 mg (0.05 ml) ranibizumab was administered at baseline, week 4 and week 8. Subsequent injections during week 12, 16, 20, 24, 36 and 48 were given if there was persistent disease activity as determined by the presence of fluid on OCT.

### Intravitreal injections

Intravitreal ranibizumab injections were administered by investigators using a strict aseptic technique under topical anesthesia in a dedicated procedure room. Intravitreal injections were performed with 29 or 30-gauge needle inserted through the inferotemporal pars plana, 4 mm posterior to the limbus in phakic eyes and 3.5 mm in pseudophakic eyes.

### Measurement of treatment outcomes

After the initiation of treatment with ranibizumab, BCVA was recorded at 4, 8, 12, 16, 20, 24, 28, 36, and 48 weeks.

The change in CRT measured by OCT, and the number of injections of ranibizumab required were also evaluated in this study. OCT, fundus photography and fluorescein angiography (FA) were done at baseline, at weeks 12, 24 and 48.

### Outcome measures

The primary outcome measure was the improvement in BCVA from baseline until week 48. The secondary outcomes were the change in central retinal thickness, progression of avascular area measured through FA, and mean number of injections received over 48 weeks.

### Safety assessments

Physical examinations with vital signs were recorded during each visit. As part of the safety assessments, intraocular pressure (IOP) was measured during each scheduled visit and carefully monitored for any significant increase. All adverse events (AEs) including serious adverse events (SAEs) were collected and recorded.

### Statistical analysis

Descriptive statistics in terms of mean and standard deviation (SD) were used for the values of BCVA, CRT and progression of avascular area. Change in BCVA was analyzed from baseline to week 48 using paired Student t-test. CRT and avascular area obtained from FA were analyzed for change from baseline to weeks 12, 24 and 48. A subgroup analysis of BCVA was performed based on age group (18–64, 65–74, 75–84 and > 84), gender, smoking status, and previous treatment for RVO.

Data were analysed using Statistical Analysis Software (SAS® Institute Inc., USA,) Version 9.4. Continuous data variables were checked for normality of distribution using the Shapiro-Wilk test. Descriptive statistics for categorical variables were obtained as absolute frequencies and percentages, while those for continuous variables were summarized as mean ± standard deviation. Equality of variance between the two groups was assessed using the Levene test. The Student’s t-test was used to compare normally distributed data with equal variance. The Mann-Whitney test was used to compare non-normally distributed data. Inferential analysis of categorical variables was performed with the Chi-square test or Fisher’s exact test. Missing data for efficacy was imputed using the Last Observation Carried Forward (LOCF) method. Results were considered statistically significant at *p* < 0.05.

## Results

One hundred eyes of 100 patients (79 BRVO and 21 CRVO) were enrolled in this multicenter study. The mean (SD) age of the enrolled patients was 62.8 (9.36) years. At baseline visit, out of the 100 patients, 56 patients had less than 6 months, and 83 patients had less than 9 months of onset of the disease. Patients belonging to the age group of < 65 years constituted 53% of the total number and 37% of enrolled patients were in the age group 65–74 years. Of all the patients, 57% were males and 43% were females. Hypertension (66%) and diabetes mellitus (35%) were the most common medical conditions associated in these patients (Table 1).

**Table 1:** Baseline Demographics and Risk Factors (Full Analysis Set^a^)

Parameters	Details
Age in years, Mean (SD)	62.8 (9.36)
Age groups	
Between 18 and 64	53 (53%)
Between 65 and 74	37 (37%)
Between 75 and 84	9 (9%)
85 and above	1 (1%)
Gender	
Male	57 (57%)
Female	43 (43%)
Ethnicity and Race	Indian (Asian)
Risk Factors	
Hypertension	66%
Diabetes Mellitus	35%
Dyslipidemia	11%
Thyroid disease	10%
Smokers	10 (10%)

^a^*FAS* Full Analysis Set comprised all patients who provided informed consent and were treated with ranibizumab in this study. (*N* = 100)

Out of 100 eyes, 47 were right eyes. Cataract followed by glaucoma were the most common associated ocular conditions in the study eye. Majority of the patients were treatment naïve. Retinal photocoagulation was performed in 15 eyes during the period of this study.

### Change in BCVA

**Fig. 1:**
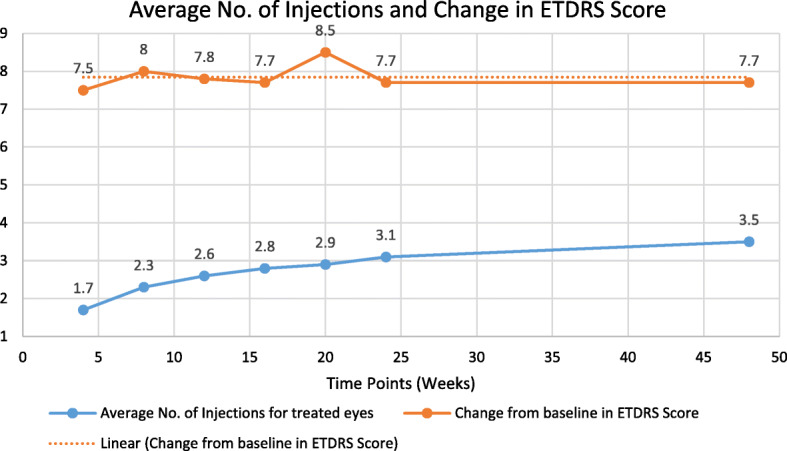
Mean Number of Injections and Visual Acuity Gain. *The maximum gain in vision from baseline occurred after the first injection and was sustained through week 48. However, the patients required further injections during the follow-up to maintain the gain in vision*

The mean (SD) BCVA was 52.8 (21.99) letters at baseline and was 62.3 (24.4) letters at week 48. The mean (SD) change in the ETDRS letter scores from baseline was 7.7 (18.38). This was statistically significant (*p* = 0.0012). The maximum gain in BCVA was achieved during week 4 and this improvement was sustained until week 48 (Fig. 1). In the treatment naïve group, visual acuity improved by 8.4 letters at the end of week 48 (*p* = 0.005), and in previously treated eyes, the BCVA improved by 5.3 letters (*p* = 0.04). The mean (SD) result of Snellen Equivalent was 6/50 (79.91) at baseline and 6/42 (82.62) at week 48.

By week 48, 43.1% patients gained > 15 letters of BCVA. (Table 2). Patients aged less than 65 years responded to injections better than older patients. There was no difference between male and female patients with respect to the outcome of the study. Both smokers and non-smokers responded well to the treatment of ranibizumab. Patients who had not received any treatment for RVO in the past responded well to the treatment (Table 3).

**Table 2:** Number (Percentage) of patients gaining or losing ETDRS letters at different visits (FAS Population)

Number of patients gaining ETDRS letters, n (%)				Number of patients losing ETDRS letters, n (%)			
	Week 12(***N*** = 86)	Week 24(***N*** = 72)	Week 48(***N*** = 65)		Week 12(***N*** = 86)	Week 24(***N*** = 72)	Week 48(***N*** = 65)
**Gain** **> = 0**	72 (83.7)	57 (79.2)	53 (81.5)	**Loss** **> = 0**	27 (31.4)	20 (27.8)	22 (33.8)
**Gain** **> = 5**	55 (64.0)	49 (68.1)	40 (61.5)	**Loss** **> = 5**	13 (15.1)	14 (19.4)	12 (18.5)
**Gain** **> = 10**	32 (37.2)	32 (44.4)	29 (44.6)	**Loss** **> = 10**	8 (9.3)	12 (16.7)	9 (13.8)
**Gain** **> = 15**	28 (32.6)	31 (43.1)	28 (43.1)	**Loss** **> = 15**	8 (9.3)	11 (15.3)	6 (9.2)

Percentage was calculated using “N” at each visit; *N* Total number of patients in full analysis set at each visit; *n* Number of patients with gaining or losing ETDRS Letters;

*ETDRS* Early Treatment Diabetic Retinopathy Scale

**Table 3:** Mean Change in ETDRS in BCVA, Retinal Thickness During the Study

		ETDRS in BCVA				Retinal Thickness			
Subgroup		Baseline (***N*** = 100) Mean (SD)	Week 48 (***N*** = 65) Mean (SD)	Change from Baseline	***p***-Value	Baseline (***N*** = 100) Mean (SD)	Week 48 (***N*** = 65) Mean (SD)	Change from Baseline	***p***-Value Change from Baseline ***p***-Value
**Age (years)**	**18–64**	54.6 (20.83) *n* = 53	67.9 (20.19) *n* = 36	10.6 (18.47) *n* = 36	0.0016	464.5 (218.03) *n* = 53	264.8 (127.57) *n* = 33	161.9 (267.60) *n* = 33	0.0015
	**65–74**	52.7 (21.55) *n* = 37	62.0 (19.86) *n* = 22	7.7 (13.70) *n* = 22	0.0156	501.2 (203.25) *n* = 36	317.4 (240.69) *n* = 17	154.6 (259.77) *n* = 16	0.0310
	**75–84**	47.1 (28.19) *n* = 9	37.5 (42.30) *n* = 6	−9.5 (27.06) *n* = 6	0.4291	493.9 (280.42) *n* = 9	307.2 (161.36) *n* = 5	273.0 (214.59) *n* = 5	0.0466
	**> = 85**	11.0 *n* = 1	20.0 *n* = 1	9.0 *n* = 1	–	408.0 *n* = 1	–	–	–
**Gender**	**Male**	48.9 (23.83) *n* = 57	58.8 (27.42) *n* = 36	6.8 (17.45) *n* = 36	0.0246	487.3 (218.94) *n* = 56	254.6 (119.33) *n* = 33	198.7 (225.43) *n* = 32	< 0.0001
	**Female**	58.1 (18.24) *n* = 43	66.7 (19.61) *n* = 29	8.8 (19.72) *n* = 29	0.0232	470.4 (214.88) *n* = 43	330.4 (224.02) *n* = 22	− 128.3 (301.35) *n* = 22	0.0589
**Smoking Status**	**Smoker**	48.8 (29.20) *n* = 10	77.8 (5.08) *n* = 6	13.7 (9.07) *n* = 6	0.0141	494.2 (266.86) *n* = 10	242.5 (77.94) n = 6	− 254.5 (401.63) *n* = 6	0.1813
	**Non-smoker**	53.3 (21.20) *n* = 90	60.7 (25.04) *n* = 59	7.1 (19.01) *n* = 59	0.0057	478.3 (211.58) *n* = 89	290.1 (179.29) *n* = 49	− 159.5 (239.33) *n* = 48	<.0001
**Previous treatment for RVO**	**Not treated**	53.4 (20.07) *n* = 79	62.8 (24.06) *n* = 50	8.4 (20.39) *n* = 50	0.0052	501.9 (225.96) *n* = 79	288.9 (191.19) *n* = 43	−187.9 (280.10) *n* = 43	<.0001
	**Laser treatment**	54.0 (24.23) *n* = 4	51.5 (32.23) *n* = 4	2.5 (8.66) *n* = 4	0.6042	454.8 (148.30) *n* = 4	306.7 (62.01) *n* = 3	122.7 (228.71) *n* = 3	0.4510
	**Previous anti-VEGF therapy**	50.8 (28.58) *n* = 21	60.6 (26.27) *n* = 15	5.3 (9.01) *n* = 15	0.04	393.3 (147.28) *n* = 20	270.8 (65.49) *n* = 12	100.0 (135.69) *n* = 11	0.0346

*N* Total number of patients, *n* Number of patients with available data

*ETDRS* Early Treatment Diabetic Retinopathy Study; *BCVA* Best Corrected Visual Acuity

### Change in CRT

**Fig. 2:**
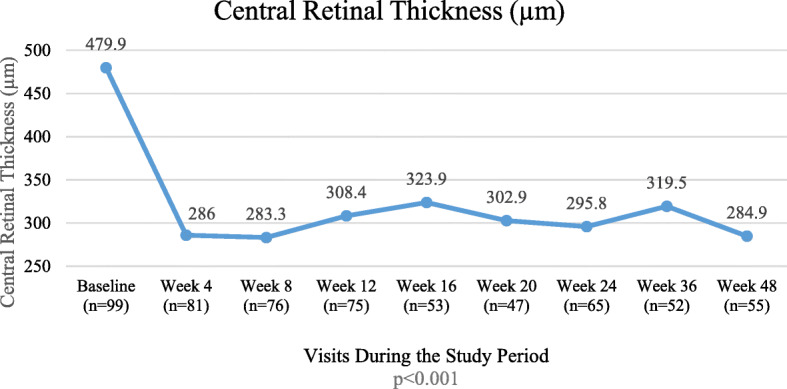
Mean Change in Central Retinal Thickness Measured by Optical Coherence Tomography for the Selected Eye. After the initial 3 loading doses, there is a gradual worsening of macular edema between weeks 8 and 16, suggesting under-treatment during this period. n: Number of patients with available recordings of CRT

There was a significant decrease in CRT during week 12, week 24 and week 48. After the first IVI of ranibizumab, there was a substantial decrease in mean CRT observed at week 4 (Fig. 2) and the decrease in thickness was maintained throughout the study. The mean (±SD) retinal thickness decreased from 479.9 (±216.25) μm at baseline to 284.9 (±171.35) μm at Week 48. In the treatment naïve group, there was a significant reduction in CRT of 187.9 μm (*p* < 0.0001). In the eyes, previously treated with anti-VEGF agents, the CRT was reduced by 100 μm (*p* = 0.0346).

### Number of injections

The mean number of injections received by the patients through week 48 was 3.5. The mean number of injections plotted against gain in BCVA is depicted in Fig. 1.

### Changes in FAZ

FA was performed to evaluate the Foveal Avascular Zone (FAZ). The diameter (mm) and area (mm^2^) of FAZ were recorded at baseline, and at weeks 12, 24 and 48 (Table 4). Treatment with ranibizumab did not show significant decrease in the diameter and area of FAZ.

**Table 4:** Mean Change in Avascular Zone by Fluorescein Angiography During the Study

	Mean (SD) of FAZ Diameter (mm)				Mean (SD) of FAZ Area (mm^**2**^)			
	Baseline ***n*** = 46	Week 12 n = 24	Week 24 ***n*** = 14	Week 48 ***n*** = 16	Baseline ***n*** = 46	Week 12 ***n*** = 24	Week 24 ***n*** = 14	Week 48 ***n*** = 17
**Mean (SD)**	0.81 (0.396)	0.71 (0.363)	0.83 (0.519)	0.70 (0.389)	0.46 (0.322)	0.33 (0.246)	0.40 (0.408)	0.33 (0.212)
**Change from Baseline**	–	−0.01 (0.361)	0.20 (0.343)	0.10 (0.440)	–	−0.04 (0.214)	0.07 (0.297)	−0.00 (0.203)
**95% Confidence Interval**	–	−0.16, 0.14	0.00, 0.40	−0.14, 0.33	–	−0.13, 0.05	− 0.10, 0.25	−0.11, 0.10
*p*-value*	–	0.8964	0.0477	0.3835	–	0.3713	0.3665	0.9370

*p-value calculated using unpaired t-test

*FAZ* Foveal Avascular Zone -Diameter (in millimeters); *FAZ* Foveal Avascular Zone-Area (in squared millimeters)

### Follow up

Out of the 100 patients enrolled in the study, 65 patients completed the follow-up at 48 weeks, and 35 discontinued or were lost to follow-up. Two patients withdrew consent, 3 patients discontinued due to administrative reasons, and 1 patient discontinued due to unsatisfactory therapeutic effect.

### Safety outcomes

A total of 13 AEs were reported in 8 patients. Of these, 8 AEs (in 6 patients) were ocular, and 5 AEs (in 3 patients) were non-ocular. Out of the 13 AEs, 12 were treatment emergent adverse events. Six AEs were mild, one AE was moderate, and 6 AEs were severe in intensity. (Table 5 and Table 6).

**Table 5:** Ocular Adverse Events and Serious Adverse Events

	FAS(***N*** = 100)
Patients who had an Adverse Event, n (%)^a^	8 (8)
Total no. of Adverse Event, E	13
Total no. of TEAEs	12
Severity, n (%) ^b^	
Mild	6 (46.2)
Moderate	1 (7.7)
Severe	6 (46.2)
Site of AE, E	
Non-ocular	5
Left eye	4
Right eye	3
Both eyes	1
Relationship to study drug or ocular injection, n (%) ^b^	
Not suspected	13 (100.0)
Action taken, n (%) ^b^	
No action taken:	2 (15.4)
Concomitant medication taken	6 (46.2)
Non-drug therapy given:	4 (30.8)
Hospitalization/prolonged:	1 (7.7)

^a^ Percentage was calculated by using Full Analysis Set

^b^ Percentage was calculated by using total number of adverse drug reactions

*TEAE* New or worsened AE after start of ranibizumab; *N* Total number of patients; *n* Number of patients with available data; *E* Number of events – Adverse events were coded using MedDRA version 19.0

**Table 6:** Summary of Adverse Events in Patients Receiving Ranibizumab

Ocular Adverse Events (E = 8)		Non-ocular Adverse Events (E = 5)	
Angle Closure Glaucoma	1	Neck Pain	1
Cataract	1	Dizziness	1
Pigment Dispersion Syndrome	1	Hemorrhagic Stroke	1
Vitreous Hemorrhage	1	Headache	1
III Nerve Paralysis	1	Dog Bite	1
Intraocular Pressure Increased	2		
Neovascularization	1		

*n* = 8; *n* Number of patients with adverse events; *E* Number of events

A total of 6 serious adverse events (SAEs) were reported in 5 patients. As part of safety IOP was measured during the study and change in IOP was obtained for the study eye. Three patients had increased IOP. Hemorrhagic stroke and III nerve paralysis were reported in 2 patients. Relationship of the SAEs to the study medication was not suspected. None of the patients developed endophthalmitis.

## Discussion

This was an open label study conducted for 48 weeks in patients with ME secondary to RVO. The eligibility criteria were not very stringent to accommodate more patients in the study. Our results reflect real-life situation in clinical practice and have significant external validity.

The improvement in BCVA from baseline was evident as early as week 4 and the maximum increase in BCVA was at week 20. At week 48, an improvement of 7.7 letters was seen, which was much less than the improvement noted in other pivotal trials of ranibizumab for treatment of RVO [7, 8]. This is likely due to under treatment of patients in the real world, leading to suboptimal outcome. Another reason for the poor improvement in vision in our patients could be the fact that 21% of patients enrolled in this study had received prior anti-VEGF injections. Patients who have received prior treatment may be in the plateau phase of gain in vision and may not gain further vision with a greater number of injections. A gain of 15 or more ETDRS letters was achieved in 43.1% of our patients. While this was substantial, it is less than the proportion of patients gaining 15 letters that are reported in the randomised trials [7–9]. However, results of our study are better than some of the results reported in the literature [10].

In our study, the average number of injections received over a period of 48 weeks was 3.5. As this was an observational phase 4 study, the treatment regimen was as per the clinical judgment of the treating physician. The number of injections in the real world depends on various factors related to the patient like visual benefits versus expectations, out of pocket expense, caregiver burden, frequency of visits, reluctance to be injected, and adherence to treatment. All these factors lead to potential under-treatment. Similarly, other real world studies have shown annual injection frequency ranging from 3 to 5 in RVO [11–16].

In our study, ranibizumab reduced the CRT by approximately 40% in as early as 4 weeks and this reduction was maintained through week 48. This negative correlation between CRT and BCVA has been substantiated by other studies [7, 8, 17, 18]. Our study showed that patients were probably under-treated between weeks 8 and 16, when the macular thickness showed an increasing trend. This may be due to a tendency among practitioners to be less aggressive after the first 3 injections, leading to less than optimal final visual outcome.

We performed a subgroup analyses to look at the impact of different age groups, smoking status and previous treatment for RVO on the treatment outcome. Age may play an important role while selecting the patients with RVO for treatment with ranibizumab and the outcome of treatment with ranibizumab may be better in younger patients with RVO. Patients with age less than 65 years showed significant improvement in visual gain during the treatment period.

There was no difference in improvement in vision among smokers and non-smokers. However, very few smokers were part of the analysis and it may be difficult to draw a definitive conclusion on the impact of the smoking on BCVA. During this study, no new or unexpected ocular or non-ocular safety events were identified. Elevation in IOP can be expected during the treatment with ranibizumab, and our study had 2% of patients developing glaucoma which was well controlled with medication [19]. However, in case of patients with preexisting glaucoma or ocular hypertension, this may be of concern.

The possibility of endophthalmitis and retinal detachment following IVI of ranibizumab are of concern [7, 20]. No such ocular adverse events occurred in our study. The findings from our study were comparable to other similar studies and there were no new adverse events reported.

IVI ranibizumab has been compared with laser therapy in patients with RVO and reported to be more effective [21]. In the treatment naïve group, visual acuity improved by 8.4 letters at the end of week 48, which was inferior to the randomized controlled trials. Unlike randomized controlled trials, this real-world observational study has limitations such as lack of homogenous group of patients with stringent eligibility criteria. The sample size is limited for this study. The results can be assumed to give a fair indication of the efficacy of ranibizumab in RVO in the real-world with less than optimal injections and follow-up. However, safety signals require a much larger sample size. Secondly, differentiation of CRVO and BRVO was not done in this study and no data were collected under these two subgroups. Hence, no subgroup analysis could be performed to understand the efficacy of ranibizumab in these two conditions.

## Conclusions

In this real-world prospective study on the safety and efficacy of ranibizumab in RVO, a significant improvement in BCVA was observed as assessed by gain in ETDRS letters. There were no new safety signals in this study. However, under-treatment lead to sub-optimal overall visual gain.

## Data Availability

The datasets used and/or analysed during the current study are available from the corresponding author on request.

## Abbreviations

AE: Adverse event
BCVA: Best corrected visual acuity
CRT: Central retinal thickness
DCGI: Drug controller general of India
ETDRS: Early treatment of diabetic retinopathy study
FA: Fluorescein angiography
FAZ: Foveal avascular zone
IOP: Intraocular pressure
IVI: Intravitreal injection
LOCF: Last observation carried forward
ME: Macular edema
μm: Micrometer
RCT: Randomized controlled trial
RVO: Retinal vein occlusion
SD: Standard deviation
SD-OCT: Spectral domain optical coherence tomography
SAE: Serious adverse event
USFDA: United States food and drug administration
VEGF: Vascular endothelial growth factor
